# Association of PTEN Gene SNPs rs2299939 With PFS in Patients With Small Cell Lung Cancer Treated With Early Radiotherapy

**DOI:** 10.3389/fgene.2020.00298

**Published:** 2020-04-23

**Authors:** Chunbo Wang, Depeng Yang, Xiaoqing Zhang, Xiaohan Zhang, Lijun Yang, Pingping Wang, Wenyang Zhou, Huaxin Li, Yiqun Li, Huan Nie, Yu Li

**Affiliations:** ^1^Department of Radiotherapy, The Third Affiliated Hospital of Harbin Medical University, Harbin, China; ^2^School of Life Sciences and Technology, Harbin Institute of Technology, Harbin, China

**Keywords:** SCLC, PFS, radio-chemotherapy, rs2299939, PTEN

## Abstract

Lung cancer has higher morbidity and mortality than most cancers. It is common that there are some phenomenons of secondary drug resistance, radiotherapy resistance and poor prognosis during the treatment of small cell lung cancer (SCLC). Recent studies revealed that the single-nucleotide polymorphisms (SNPs) are associated with the curative effect among patients with the same pathological type and stage. Our study analyzed the start time of radiotherapy and the relationship between PTEN gene rs2299939 polymorphisms and survival time among 116 SCLC patients. The results showed that early radiotherapy significantly improved the time of survival in patients compared with late radiotherapy (**P** = 0.029). Simultaneously, the study found that patients with the rs2299939 AA genotype showed significant sensitivity to both early and late radiotherapy, but early radiotherapy is better. The median survival time of CC genotype patients was 12 months in the early radiotherapy group while it was 9 months in the late radiotherapy group, thus recommending early radiotherapy among these patients. In addition, it was found that rs2299939 could regulate the expression of related genes in peripheral blood and lung tissues by eQTL analysis. This study revealed that the early radiotherapy could prolong the PFS of SCLC and shall be performed in SCLC treatment.

## Introduction

Lung cancer is a type of common malignancy with high morbidity and mortality, seriously threatening human health (Torre et al., 2015). One in four cancer deaths is caused by lung cancer, which is regarded as the biggest killer among men and women. Small cell lung cancer (SCLC), accounting for about 15–20% of all lung cancer, is characterized by high malignancy, rapid growth, fast metastasis, and poor prognosis (Jackman and Johnson, 2005; Donington et al., 2012). Five-year survival rate is only 3–8%, and median survival following diagnosis is estimated to be 8–20 months (Imperatori et al., 2010). Radiotherapy combined with chemotherapy is still the main therapy for patients with SCLC, although the innovation of precise medical treatment and targeted drug treatment technology has reduced the mortality rate of lung cancer. Even though SCLC is sensitive to radio-chemotherapy and has high remission rate at first treatment, secondary drug resistance and radiotherapy resistance are also common (Leo and Pastorino, 2003; Rossi et al., 2013).

The key factor of radiotherapy and chemotherapy killing cells is to cause DNA damage. However, DNA damage is not always fatal because of the DNA damage repair process (Helleday et al., 2008). For example, PTEN is an inhibitor of the PI3K–AKT–mTOR pathway, and its inactivation leads to accelerating SCLC growth and increasing metastases in a Trp53 and Rb1-inactivated deleted system (Cui et al., 2014).

In this DNA damage repair process, some studies have shown that functional single-nucleotide polymorphisms (SNPs) could affect the expression of DNA damage repair-related genes (Czarny et al., 2017). SNP has been widely used as a biomarker of individual differences (Yoon et al., 2010; Lee et al., 2013). Many studies show that the risk of lung cancer, therapeutic efficacy, and prognosis is tightly correlated with SNP loci of many genes (Sato et al., 2011; Han et al., 2013, 2014; Yoon et al., 2014; Tang et al., 2015). Some studies focused on the DNA damage repair-related genes, such as XRCC1 (Tiseo et al., 2013), ERCC1 (Ren et al., 2012), TP53, PARP1 (Shiraishi et al., 2010), and XPD (Santarpia et al., 2017) in NSCLC, etc. Our group has found that rs12334811, rs11615, and rs2299939 in DNA repair pathway genes might regulate the expression and affect the DNA damage repair and thereby impact the efficacy of radio-chemotherapy and the survival time of NSCLC (Wang et al., 2016).

There are already many studies focusing on DNA damage repair-related genes and radio-chemotherapy in NSCLC, but several studies on SCLC have been conducted. Rs7963551 (RAD52) (Li et al., 2016), rs7003908 (XRCC7) (Singh et al., 2018), and MTH1 (Kohno et al., 2006) were involved in susceptibility to SCLC. rs10774474 (RAD52) played a key role in the response to platinum-based chemotherapy (Zhang et al., 2017). rs2267437 (XRCC6) (Singh et al., 2018) and rs942190 (TDP1) (Lohavanichbutr et al., 2017) were associated with survival time among SCLC patients. These results showed that the genetic variations involved in DNA repair pathways are related with SCLC susceptibility, treatment, and survival time. However, there is no idea about the relation between the PTEN polymorphisms and SCLC. Although some progresses have been made in the radio-chemotherapy of SCLC, problems still exist in clinical application, such as the timing of start radiotherapy and whether the early or the late stage is better. There is still a huge difference in clinical efficacy and side effects even in patients with the same pathological type and age. Individual genetic factors may contribute to the difference, but the impact of this factor is not often considered in clinical practice.

This study analyzed the different timing of radiotherapy start among 116 patients with SCLC and the polymorphisms of PTEN (rs2299939), which is related to DNA damage repair. We found that early radiotherapy could significantly improve the progression-free survival (PFS) of patients, which is a survival index. In addition, the AA genotype of rs2299939 has the best therapeutic effect. The CC genotype was suggested to receive early radiotherapy, while the CA genotype has no significant difference between early and late radiotherapy. This research revealed that the start time of radiotherapy is significant to prolong the PFS of SCLC. At the same time, the results will increase the understanding of the relation between the polymorphisms of DNA damage repair gene and SCLC, and it will provide new guidance for clinical treatment on SCLC of different genotypes.

## Materials and Methods

### Sample Collection and Treatment

The research was approved by the Ethical Committee of the Tumor Hospital of Harbin Medical University. SCLC patients were recruited between March 2010 and March 2013 in the hospital and provided with informed consent. Clinical data and fasting venous blood were collected. A total of 116 patients were included in this study. All patients except six cases were followed up until August 2013. The first-line treatment was EP (etoposide + cisplatin) or EC (etoposide + carboplatin) chemotherapy, which was repeated every 3 weeks for four to six cycles. Radiotherapy was three-dimensional intensity modulated radiation therapy with conventional fractionation. The total dose was 50–60 Gy/27–32 times/37–45 days. Early radiotherapy group started after 1–2 cycles of chemotherapy while late radiotherapy group started after 3 cycles. PFS, remission time after treatment, progression mode after first-line treatment, and sensitivity to radio-chemotherapy (effectiveness) are included in follow-up records.

### DNA Extraction and SNP Detection

The methods of DNA extraction and SNP detection were similar to the previous study. In brief, DNA was extracted from patients’ peripheral blood by saturated phenol method and working concentration was 50 ng/μl for each DNA sample. SNP genotyping was detected by CapitalBio (Beijing, China) using the Sequenom MassARRAY platform (Sequenom, San Diego, CA, United States). Data were processed and analyzed by Sequenom MassArray TYPER 4.0 software.

### eQTL Analysis

We evaluated the distribution of genetic variation in promoter, enhancer, and DNase I hypersensitive sites from different tissues and cells using HaploReg v4.1^1^. This study predicts distribution of DNA genetic variation in different promoter and enhancer regions based on the core 15-state model.

### Statistical Analysis

Bivariate Logistic Regression Analysis was used for multivariate analysis, χ^2^ analysis was used for univariate analysis, and the Kaplan–Meier method was used for survival analyses. Analyses were performed using SPSS and R version 3 software packages. All *P* values were two-sided.

## Results

### Association of Radiotherapy Starting Time With SCLC Patients’ Survival

Based on general clinical factors, 116 SCLC patients were divided into two groups according to starting time (Table 1). Early radiotherapy means that radiotherapy begins after the first or second cycle of chemotherapy, while late radiotherapy begins after the third cycle of chemotherapy. van Meerbeeck regarded 90 days as the critical point of length of remission. The length of remission period should be divided into less than or equal to 90 days and greater than 90 days (Van Meerbeeck et al., 2011). The effect of “the 90 days after the therapy” was used as an independent variable analyzed by bivariate logistic regression. The results showed that there were significant differences between early and late radiotherapy (*P* < 0.05), indicating that the starting time of radiotherapy could significantly affect the remission time after first-line treatment.

**TABLE 1:** Analysis of clinical characteristics and remission time among 116 patients with small cell lung cancer.

**Characteristics**		***N***	**Remission time > 90 days**	**Remission time ≤ 90 days**	**Univariate analysis *P*-value**	**Multivariate analysis *P*-value**
Sex	Male	76	44	32		
	Female	40	25	15	0.631	0.517
Age	≥58 years	62	41	21		
	<58 years	54	28	26	0.133	0.868
Smoking status	Non-smoker	61	39	22		
	Smoker	55	30	25	0.346	0.077
Clinical stage	Limited	72	48	24		
	Extensive	44	21	23	0.053	0.007
The timing of radiotherapy	Early stage	36	31	5		
	Late stage	80	38	42	0.000	0.015
Chemosensitivity	Valid	86	58	28		
	Invalid	30	11	19	0.005	0.002
Progressive manner	Stability	17	13	4		
	Recrudesce	27	19	8		
	Brain metastases	30	17	13		
	Other	42	16	26	0.024	0.569

The relationship between the starting time of radiotherapy and the proportion of recurrence and metastasis in SCLC patients was further analyzed (Table 2). The results showed that about 27.8% of early radiotherapy patients had no recurrence or metastasis and about 72.2% had recurrence or metastasis in 90 days after operation, while among late radiotherapy patients, only 8.8% of patients had no recurrence or metastasis and 91.2% had recurrence or metastasis in 90 days after operation (*P* = 0.035). These data indicated that early radiotherapy was of great significance for the recurrence and metastasis of SCLC.

**TABLE 2:** The influence of radiotherapy intervening time on the progressive manner after treatment.

		**Progressive manner after treatment**					**Total**
			
		**No recurring metastasis**	**Recurrence**	**Brain metastases**	**Other metastasis**	**Uncertain**	
Early radiotherapy	*N* (%)	10 (27.8%)	7 (19.4%)	9 (25.0%)	8 (22.2%)	2 (5.6%)	36 (100.0%)
Late radiotherapy	*N* (%)	7 (8.8%)	20 (25.0%)	21 (26.2%)	28 (35.0%)	4 (5.0%)	80 (100.0%)
Total	*N*	17	27	30	36	6	116

*χ^2^ = 3.627, *P* = 0.035.*

**FIGURE 1:**
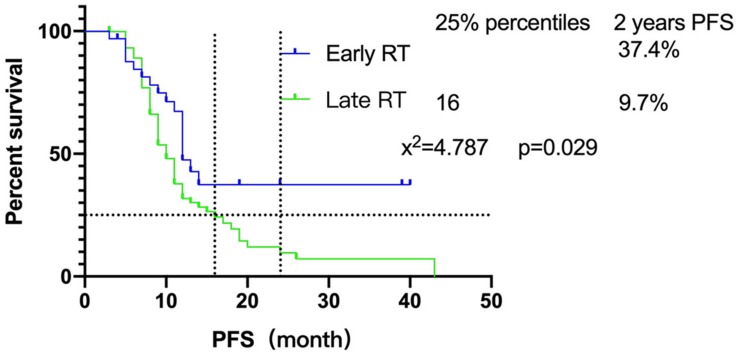
The influence of radiotherapy intervening time on PFS between two groups.

Furthermore, we focused on the relationship between radiotherapy start time and PFS. The survival of patients showed that there was a significant difference between early radiotherapy and late radiotherapy (*P* = 0.029) (Figure 1). Until the end of follow-up, the 25% PFS of late radiotherapy patients was 16 months while the early radiotherapy patients had not been able to reach the 25% PFS. Sixteen of 33 (48.5%) early radiotherapy patients are progression-free, while 14/75 (18.7%) of the late radiotherapy patients are progression-free. In addition, the 2-year PFS of early and late radiotherapy patients was 37.4 and 9.7%, respectively, which indicated that early radiotherapy was better than late radiotherapy for SCLC patients.

### Association of Radiotherapy Starting Time With Chemotherapy

**FIGURE 2:**
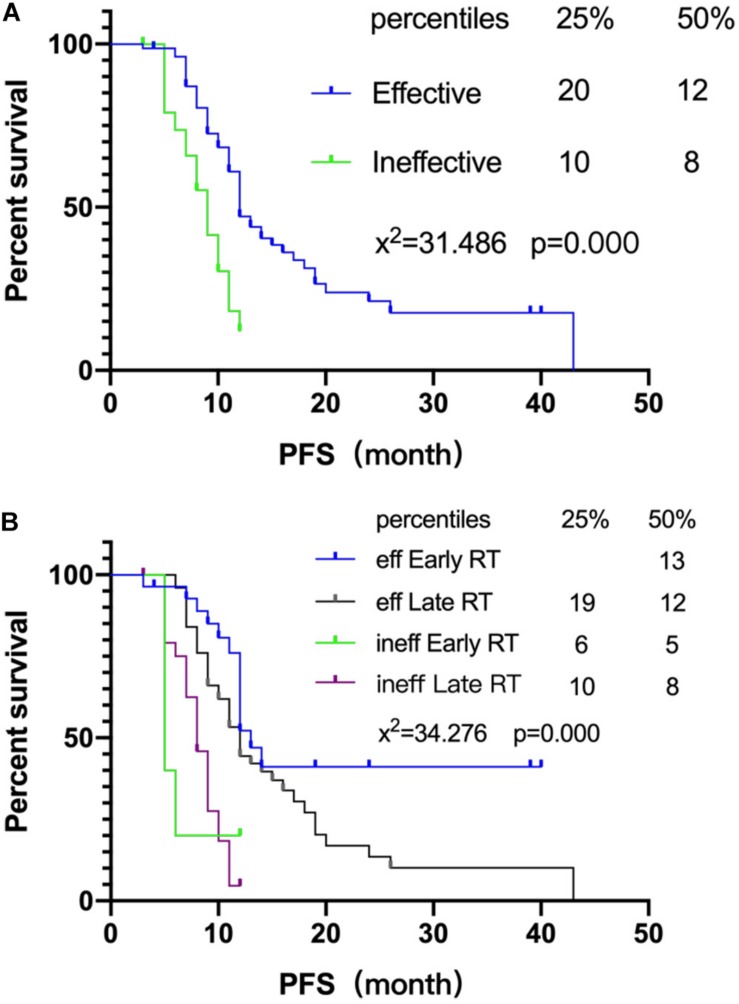
Association of radiotherapy starting time with chemotherapy. **(A)** Survival curves of chemotherapy efficiency and PFS. **(B)** Survival curves of chemotherapy efficiency and PFS of different radiotherapy intervening groups.

Further analysis was performed to analyze the relation between the radiotherapy starting time and association of radiotherapy starting time with chemotherapy. The chemotherapy sensitivity of 116 SCLC patients was analyzed in this study and 80 patients were found sensitive (reduced lesion after chemotherapy, lesion narrowing more than 30% after chemotherapy according to recist 1.1). Early or late radiotherapy patients were divided according to the sensitivity to chemotherapy. As for the chemo-insensitive group, the non-recurrence rate of chemotherapy-insensitive patients is similar between the early or late radiotherapy group. However, for the chemo-sensitive group, early radiotherapy could significantly improve the non-recurrence rate compared with the late group, 34.5% (10/29) vs. 11.8% (6/51) (Table 3). At the same time, chemotherapy sensitivity could also affect PFS significantly (Figure 2A). Early radiotherapy could further improve the PFS of chemo-sensitive patients. PFS of 25% of early radiotherapy patients has not been achieved while that of late radiotherapy was 19 months *P* < 0.05 (Figure 2B). The study also found that for chemo-insensitive patients, early or late radiotherapy did not change the overall survival trend; PFS of 25% of patients are 6 months and 10 months, respectively. Therefore, chemotherapy sensitivity determines the overall therapeutic effect of patients. Early radiotherapy can significantly increase the rate of non-recurrence and metastasis, suggesting that early radiotherapy as a local treatment can reduce the recurrence rate and further reduce the metastasis rate of chemo-sensitive patients.

**TABLE 3:** Relation between chemosensitivity and progressive manner of different radiotherapy intervening groups.

**Groups of radiotherapy intervening time and chemosensitivity**		**Progressive manner after treatment**				**Total**
			
	**No recurring metastasis**	**Recurrence**	**Brain metastases**	**Other metastasis**		
Chemotherapy-sensitive group	Early radiotherapy	10 (34.5%)	6 (20.7%)	7 (24.1%)	6 (20.7%)	29 (100.0%)
	Late radiotherapy	6 (11.8%)	11 (21.6%)	18 (35.3%)	16 (31.4%)	51 (100.0%)
Chemotherapy-insensitive group	Early radiotherapy	0 (0.0%)	1 (20.0%)	2 (40.0%)	2 (40.0%)	5 (100.0%)
	Late radiotherapy	1 (4.0%)	9 (36.0%)	3 (12.0%)	12 (48.0%)	25 (100.0%)
Total	Early radiotherapy	10	7	9	8	34
	Late radiotherapy	7	20	21	28	76

*Six cases had uncertain progressive manner among 116 patients.*

### PFS Analysis of the Relationship Between rs2299939 SNPs and Early or Late Radiotherapy

**FIGURE 3:**
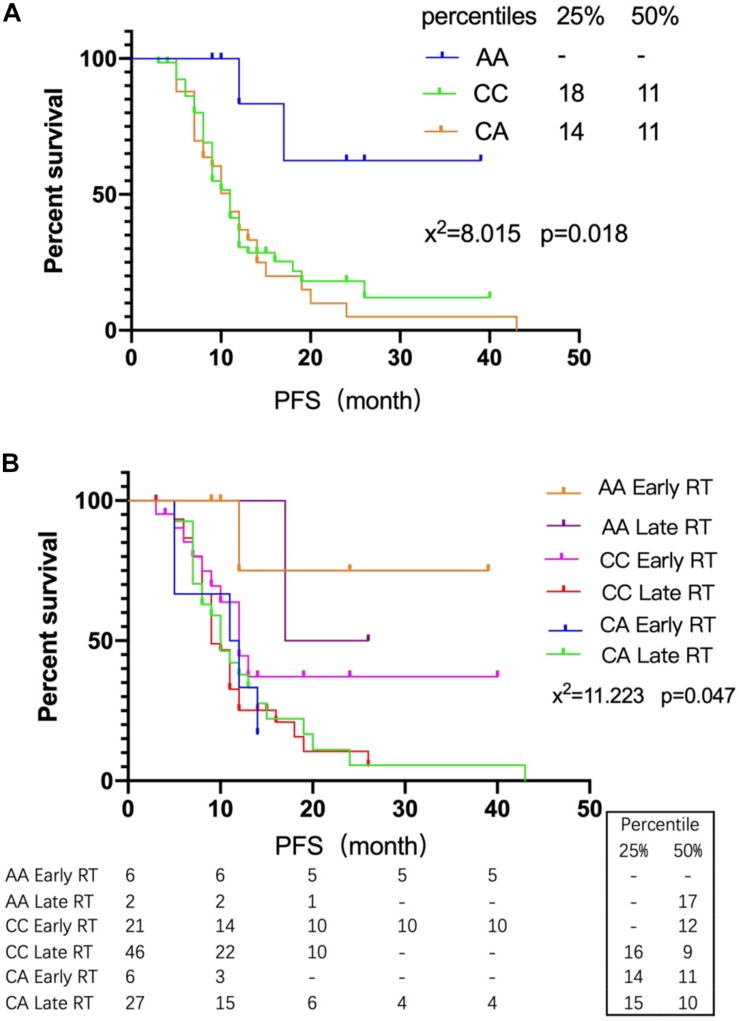
The relationship between rs2299939 SNPs and early or late radiotherapy. **(A)** Survival curves of different genotypes of rs2299939 and PFS. **(B)** Survival curves of different genotypes of rs2299939 with various radiotherapy intervening times and PFS.

The genotype distribution of rs2299939 (PTEN) and PFS was analyzed in 116 SCLC patients (Table 4). The results showed that PFS of AA genotype patients was the longest and did not reach the median PFS until follow-up surgery. The median PFS of CA and CC genotype patients was 11 months (*P* = 0.018), as shown in Figure 3A.

**TABLE 4:** The genotype distribution of rs2299939.

	***N***	**%**
CC	72	62.1
AA	10	8.6
CA	34	29.3
Total	116	100.0

The SCLC patients were grouped according to different genotypes of rs2299939. The PFS differences between early and late radiotherapy patients were studied. The results showed that the AA genotype of rs2299939 had longer PFS in both early and late radiotherapy, and only 1/6 of early radiotherapy patients had progressed while 1/2 of late radiotherapy patients had progressed. About 83.3% of the early radiotherapy patients did not reach the median PFS until follow-up time (Figure 3B). The results indicated that for rs2299939, patients with the AA genotype had longer PFS and early radiotherapy could significantly improve the survival rate, which had important guiding significance for treatment options.

Simultaneously, there was a prominent difference between early and late radiotherapy patients with the CC genotype of rs2299939 (*P* = 0.09). The curve diverged sharply around 12 months. The median PFS of the CC genotype in early radiotherapy was 12 months, while it was 9 months in late radiotherapy. For 25% PFS, patients with the CC genotype in early radiotherapy did not reach PFS, which means more patients were alive until they are followed up, while the late radiotherapy is 16 months. To some extent, it also indicated that compared with late radiotherapy, the effect of early radiotherapy on rs2299939 CC genotype was better. It suggested that patients with the rs2299939 CC genotype should also receive early radiotherapy as soon as possible. In addition, the results also showed that there was no significant difference in PFS between the early and late radiotherapy groups of the rs2299939 CA genotype. This result indicated that if the patient was a CA genotype, late start of radiotherapy might be considered in order to alleviate the side effects of radiotherapy.

Besides accomplishing PFS analysis of relationship between different genotypes of rs2299939 and early or late radiotherapy, we also studied the sensitivity of different rs2299939 genotypes to chemotherapy (Table 5). The therapeutic effect of CC genotype chemo-sensitive patients revealed that early radiotherapy could increase the effect compared with the chemo-insensitive group. The cases of CA are not enough for statistical analysis and also revealed that early radiotherapy is better. The results also showed that the AA genotype was sensitive to chemotherapy.

**TABLE 5:** Effect of chemotherapy efficiency on different chemotherapy intervening times and genotypes.

**Genotype**	**Chemosensitivity**	**The timing of radiotherapy**	**Remission time after first-line treatment**		
			
			**≤90**	**>90**	**Total**

			***N* (%)**	***N* (%)**	***N***
CC	Sensitive	Early stage	3(15.0%)	17(85.0%)	20
		Late stage	12(37.5%)	20(62.5%)	32
	Insensitive	Early stage	0(0%)	3(100.0%)	3
		Late stage	13(76.5%)	4(23.5%)	17
AA	Sensitive	Early stage	1(14.3%)	6(85.7%)	7
		Late stage	0(0%)	3(100.0%)	3
	Insensitive	Early stage	0(0%)	0(0%)	0
		Late stage	0(0%)	0(0%)	0
CA	Sensitive	Early stage	0(0%)	4(100.0%)	4
		Late stage	12(60.0%)	8(40.0%)	20
	Insensitive	Early stage	1(50.0%)	1(50.0%)	2
		Late stage	5(62.5%)	3(37.5%)	8

### Functional Analysis of rs2299939

**FIGURE 4:**
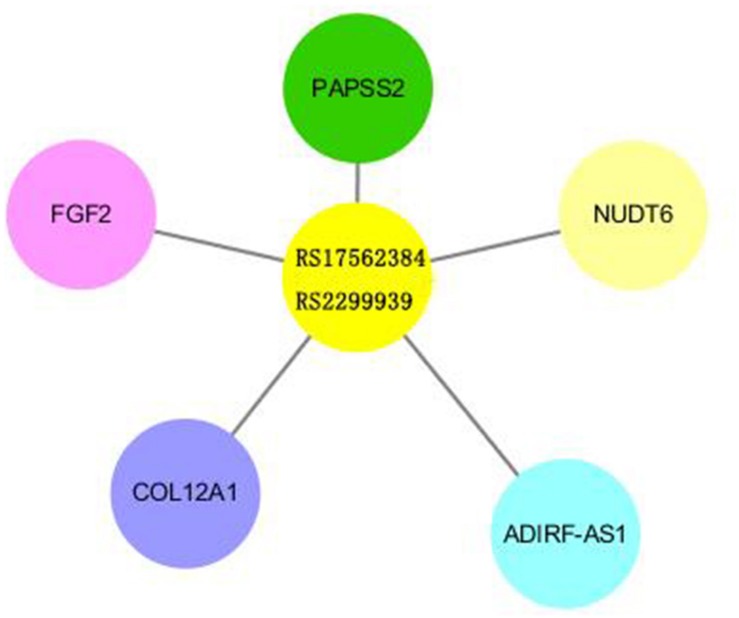
Results of eQTL analysis of rs2299939/rs17562384. The length of the bars revealed the relativity between SNP and Exp genes.

Rs2299939 is an Intron Variant of PTEN gene on chromosome 10. The function of rs2299939 was predicted using HaploReg v4.1 database and the results indicated that rs2299939 may regulate gene expression in lung tissue (Supplementary Table S1). Based on that, we selected the real genotype and expression data from the Phenoscanner database for eQTL analysis. As rs2299939 is not contained in the database, the proxy variant rs17562384 was selected to be analyzed, which is another Intron Variant of PTEN, and its location and function were similar to that of rs2299939 (*r* = 0.87, *D*′ = 0.95). The results showed that rs17562384/rs2299939 could regulate gene expression in peripheral blood, lung, and other tissues (Figure 4 and Supplementary Table S2).

## Discussion

Compared with NSCLC, SCLC has a higher degree of malignancy and faster progression and metastasis (Donington et al., 2012; Unsal et al., 2016). Although SCLC patients are sensitive to radio-chemotherapy, recurrence and metastasis always occur soon (Sas-Korczynska et al., 2013). The median survival time of SCLC patients is about 15–20 months, while that of SCLC patients in the extensive period is only 8–13 months; 2-year survival rate in the limited period is 20–40%, and that for the extensive period is only 5%. Prognosis was also worse compared with NSCLC patients (Sun et al., 2010; Byers and Rudin, 2015; Kalemkerian, 2016).

There are differences in the timing of radiotherapy for LD-SCLC according to international guidelines for lung cancer. NCCN (National Comprehensive Cancer Network) guidelines point out that synchronous radio-chemotherapy is a more standard treatment compared with sequential radio-chemotherapy and radiotherapy should be intervened as early as possible in the first or second cycles of chemotherapy. The shorter the time from the beginning of any treatment to the end of radiotherapy, the more likely it is to improve the survival time of patients (Kalemkerian et al., 2018). In the clinical guidelines of SEOM (Spanish Society of Oncology Medicine), they point out that the optimal radio-chemotherapy strategy for LD-SCLC is to start radiotherapy at the beginning of the second cycle (Domine Gomez et al., 2013). ESMO (European Society for Medical Oncology) guidelines recommend that radiotherapy should start within 30 days after chemotherapy, but for patients with poor physical condition and intolerance, radiotherapy is recommended to be delayed until the start of the third cycle (Fruh et al., 2013). So, it is important to study the advantages and disadvantages of the choice of start time for radiotherapy. In this study, multivariate analysis was made based on the 90-day progression time. All stages, chemo-sensitivity, and timing of radiotherapy start showed significant effects. Combined with the failure mode of treatment, it was found that the early radiotherapy group is more stable and the late radiotherapy group had more recurrences and metastases cases. Further survival analysis showed that PFS of the early radiotherapy group was significantly longer than that of the late radiotherapy group, which is in agreement with previous reports that show that early radiotherapy is associated with increased survival in limited-state SCLC compared with late radiotherapy (Fried et al., 2004; Pijls-Johannesma et al., 2007).

Single-nucleotide polymorphism is the third generation of molecular markers of genetic variation, which is mainly used to study the differences in susceptibility of disease and sensitivity to different medication and treatment methods. To date, studies on relationship between lung cancer and SNPs mainly focused on NSCLC, and not much on SCLC (Knoefel et al., 2011; Gu et al., 2012; Szondy et al., 2012; Cao et al., 2014).

The mechanism of radiotherapy and chemotherapy’s killing effect is to cause DNA damage (Chen et al., 2019). However, it is not always fatal because of the DNA damage repair process. Most DNA damage that happened in normal cells can be repaired (Helleday et al., 2008; Pourel, 2016). However, to a cancer cell, it is beneficial since it can result in tolerance to the treatment in tumor tissues, decreasing sensitivity and ultimately causing treatment failure. In conclusion, DNA damage repair-related genes affect the efficacy of radio-chemotherapy. Our study found that rs2299939 can affect the efficacy of SCLC. Rs2299939 locus belongs to the PTEN gene, which is an anti-oncogene abnormal in many tumors and plays an important part in the PI3K/PTEN/AKT/mTOR pathway. Cui et al. revealed that the PTEN is a potent suppressor of SCLC. They found that inactivation of one allele of PTEN might cause the acceleration of SCLC in Rb/p53-deleted mice (Cui et al., 2014). Toulany pointed out that the PI3K/AKT signal regulated by PTEN was a key mediator of radio-reactivity changes in tumor cells. Overactivation of the PI3K/AKT pathway was associated with tumorigenesis, progression, poor prognosis, and tolerance to treatment (Toulany and Rodemann, 2015). Our results suggested that the AA genotype of rs2299939 in SCLC was very sensitive to radiotherapy, and early radiotherapy could significantly improve the PFS. Early radiotherapy was better than late radiotherapy in the CC genotype of rs2299939. We also evaluated the distribution of genetic variation in promoter, enhancer, and DNase I hypersensitive sites from different tissues and cells using HaploReg v4.1. This study predicts that five EXP genes are related to rs2299939 based on the core 15-state model. Among these genes, PAPSS2 and FGF2 are related to the DNA damage repair process (Rieswijk et al., 2015; Dias et al., 2019). PAPSS2 plays an important part in sulfate assimilation and sulfate activation, during which it exhibits both ATP sulfurylase and APS kinase activity. FGF2 is a ligand for FGFRs, which plays a significant role in regulation of survival, division, migration, and differentiation of cells. It can also behave as a potent mitogen and induces angiogenesis. Our study found that rs17562384/rs2299939 could regulate these genes’ expression in peripheral blood, lung, and other tissues. The relationship between these genes and rs2299939 needs further study. These results indicated that rs2299939 was closely related to the treatment of SCLC. Previous studies have shown that the CA genotype of rs2299939 could reduce the risk of hepatocellular carcinoma. It revealed the association of PTEN gene polymorphisms with liver cancer risk (Du et al., 2015; Li et al., 2015). Our group previously found that the PTEN gene CC genotype of rs2299939 was sensitive to radiotherapy in adenocarcinoma and squamous cell carcinoma (Wang et al., 2016). These results suggested that rs2299939 and PTEN genes played important roles in the efficacy of radio-chemotherapy.

Overall, this study revealed that early radiotherapy is significant to prolong the PFS of SCLC. The sensitivity of rs2299939 (PTEN) to radiotherapy in 116 patients with SCLC was analyzed. It found that AA and CC genotypes of rs2299939 were sensitive to radiotherapy; meanwhile, early radiotherapy could significantly improve PFS. However, our sample size is too small, and further investigation about associations between rs2299939 and PFS are needed. This study builds a foundation for classifying patients with SNP, predicting the efficacy of radiotherapy and chemotherapy and guiding the next precise treatment in SCLC patients.

## Data Availability Statement

The datasets generated for this study can be found in BCBI BioProject ID is PRJNA579735.

## Ethics Statement

The studies involving human participants were reviewed and approved by the Ethical Committee of the Tumor Hospital of Harbin Medical University. The patients/participants provided their written informed consent to participate in this study.

## Author Contributions

CW and DY contributed equally to this work. CW, HN, and YL contributed conception and design of the study. PW and YQL organized the database. WZ and DY performed the statistical analysis. DY wrote the first draft of the manuscript. XHZ, XQZ, LY, and HL wrote the sections of the manuscript. All authors contributed to manuscript revision, read and approved the submitted version.

## Conflict of Interest

The authors declare that the research was conducted in the absence of any commercial or financial relationships that could be construed as a potential conflict of interest.

## Footnotes

1 http://pubs.broadinstitute.org/mammals/haploreg
