# Supplementary material for: Association of PTEN Gene SNPs rs2299939 With PFS in Patients With Small Cell Lung Cancer Treated With Early Radiotherapy
Source: Front Genet. 2020 Apr 23;11:298. doi: 10.3389/fgene.2020.00298 (PMC7190978; doi:10.3389/fgene.2020.00298)
Supplement: Supplementary file 1 [file Data_Sheet_1.docx]

### Association of PTEN gene SNPs rs2299939 with PFS in patients with small cell lung cancer treated with early radiotherapy

Chunbo Wang^1,2#^, Depeng Yang^2#^, Xiaoqing Zhang^2^, Xiaohan Zhang^2^, Lijun Yang^2^, Pingping Wang^2^, Huaxin Li^2^, Yiqun Li^2^, Huan Nie^2*^ Yu Li^2*^

^1^Department of Radiotherapy, Affiliated Tumour Hospital of Harbin Medical University, Harbin, China.

^2^School of Life Science and Technology, Harbin Institute of Technology, Harbin, China.

^#^These authors contributed equally to this work.

***Correspondence:**

Huan Nie

nh1212@hit.edu.cn

Yu Li

[liyugene@hit.edu.cn](mailto:liyugene@hit.edu.cn)

| Table S1 Functional prediction of rs2299939 | | | | |
| --- | --- | --- | --- | --- |
| Variant | Enhancer histone marks | DNAse | Motifs changed | Genes |
| RS2299939 | Tissues IMR90 fetal lung fibroblasts Cell Line, Primary hematopoietic stem cells, Primary hematopoietic stem cells short term culture, Primary hematopoietic stem cells G-CSF-mobilized Female, Primary hematopoietic stem cells G-CSF-mobilized Male, Fetal Heart, Fetal Lung, Left Ventricle, Ovary | BLD, LNG | Irf | PTEN |

| Table S2 Results of eqtl analysis | | | | | | | | | | |
| --- | --- | --- | --- | --- | --- | --- | --- | --- | --- | --- |
| SNP | A1 | A2 | Study | Pmid | Tissue | Exp gene | β | Se | P value | N |
| rs17562384 | C | T | GTEx | 25954001 | Prostate | ADIRF-AS1 | 0.3938 | 0.1069 | 0.0003557 | 132 |
| rs17562384 | C | T | Joehanes R | 28122634 | Whole blood | COL12A1 | 0.0068 | 0.00161 | 2.38E-05 | 5257 |
| rs17562384 | C | T | Joehanes R | 28122634 | Whole blood | FGF2;NUDT6 | 0.0224 | 0.00568 | 8.11E-05 | 5257 |
| rs17562384 | C | T | eQTLGen | eQTLGen | Whole blood | PAPSS2 | NA | NA | 4.92E-07 | 30931 |
